# Synergistic Embryotoxicity of Polycyclic Aromatic Hydrocarbon Aryl Hydrocarbon Receptor Agonists with Cytochrome P4501A Inhibitors in *Fundulus heteroclitus*

**DOI:** 10.1289/ehp.7168

**Published:** 2004-08-18

**Authors:** Deena M. Wassenberg, Richard T. Di Giulio

**Affiliations:** Nicholas School of the Environment and Earth Sciences, Integrated Toxicology Program, Duke University, Durham, North Carolina, USA

**Keywords:** α-naphthoflavone, aryl hydrocarbon receptor, benzo(*a*)pyrene, β-naphthoflavone, cytochrome P4501A, deformity, fluoranthene, *Fundulus heteroclitus*, polychlorinated biphenyls, polycyclic aromatic hydrocarbons

## Abstract

Widespread contamination of aquatic systems with polycyclic aromatic hydrocarbons (PAHs) has led to concern about effects of PAHs on aquatic life. Some PAHs have been shown to cause deformities in early life stages of fish that resemble those elicited by planar halogenated aromatic hydrocarbons (pHAHs) that are agonists for the aryl hydrocarbon receptor (AHR). Previous studies have suggested that activity of cytochrome P4501A, a member of the *AHR* gene battery, is important to the toxicity of pHAHs, and inhibition of CYP1A can reduce the early-life-stage toxicity of pHAHs. In light of the effects of CYP1A inhibition on pHAH-derived toxicity, we explored the impact of both model and environmentally relevant CYP1A inhibitors on PAH-derived embryotoxicity. We exposed *Fundulus heteroclitus* embryos to two PAH-type AHR agonists, β-naphthoflavone and benzo(*a*)pyrene, and one pHAH-type AHR agonist, 3,3′,4,4′,5-pentachlorobiphenyl (PCB-126), alone and in combination with several CYP1A inhibitors. In agreement with previous studies, coexposure of embryos to PCB-126 with the AHR antagonist and CYP1A inhibitor α-naphthoflavone decreased frequency and severity of deformities compared with embryos exposed to PCB-126 alone. In contrast, embryos coexposed to the PAHs with each of the CYP1A inhibitors tested were deformed with increased severity and frequency compared with embryos dosed with PAH alone. The mechanism by which inhibition of CYP1A increased embryotoxicity of the PAHs tested is not understood, but these results may be helpful in elucidating mechanisms by which PAHs are embryotoxic. Additionally, these results call into question additive models of PAH embryotoxicity for environmental PAH mixtures that contain both AHR agonists and CYP1A inhibitors.

Polycyclic aromatic hydrocarbons (PAHs) are important environmental contaminants that are generated by the incomplete combustion of organic compounds. PAHs enter the environment through natural sources such as forest fires and seeps in ocean floors and through anthropogenic activities, including combustion of fossil fuels and wood and petroleum refining (Douben 2003; Latimer and Zheng 2003). PAH contamination in estuarine settings originates from point sources such as municipal wastewater discharges, industrial outfalls, and oil shipping and refinery operations, and from non-point sources such as urban runoff and dry and wet depositions of atmospheric PAHs (Latimer and Zheng 2003). The ubiquity of PAH contamination at U.S. national priority sites (Superfund sites), along with their known and suspected human toxicity, has led to the listing of PAHs as eighth on the Agency for Toxic Substances and Disease Registry’s (ATSDR) priority list; 15 individual PAHs are also listed throughout the priority list of 275 entries (ATSDR 2003). Furthermore, environmental contamination by PAHs has steadily increased in recent years (Van Metre et al. 2000).

Some PAHs have impacts on early life stages of fish, including reduced growth, cranial–facial malformations, yolk sac and pericardial edema, and subcutaneous hemorrhaging (Billiard et al. 1999; Carls et al. 1999; Hawkins et al. 2002). These deformities closely resemble the “blue sac syndrome” that has been described in several fish species, including rainbow trout (*Oncorhynchus mykiss*), zebrafish (*Danio rerio*), medaka (*Oryzias latipes*), and killifish (*Fundulus heteroclitus*), exposed to certain halogenated aromatic compounds that are agonists for the aryl hydrocarbon receptor (AHR) (Chen and Cooper 1999; Elonen et al. 1998; Helder 1981; Toomey et al. 2001; Walker and Peterson 1991; Wannemacher et al. 1992). These compounds include coplanar polychlorinated biphenyls (PCBs) and 2,3,7,8-tetrachlorodibenzo-*p*-dioxin (TCDD), collectively referred to here as planar halogenated aromatic hydrocarbons (pHAHs). Some of the PAHs that induce these deformities are, like TCDD and coplanar PCBs, agonists for the AHR (Billiard et al. 2002).

The AHR is a cytoplasmic receptor whose activation initiates the transcription of a battery of genes, including the monooxygenase cytochrome P4501A (here generally referred to as CYP1A, although two CYP1As exist in mammals as well as in rainbow trout; mammalian CYP1As are referred to as CYP1A1 and CYP1A2; Hankinson 1995). The AHR pathway is similar between mammals and nonmammalian vertebrates, including fish, reptiles, and birds (Hahn 1998); however, two AHRs (AHR1 and AHR2) have been identified and characterized in several fish species, including killifish and zebrafish (Andreasen et al. 2002; Hahn et al. 1997; Karchner et al. 1999). The mechanism for the toxicity of pHAHs has been widely studied, and there are well-established positive relationships among compounds’ affinity for the AHR, their potency for CYP1A induction, and their toxicity (Guiney et al. 1997; Heid et al. 2001; Safe 1990, 1993).

The critical role of AHR in pHAH toxicity has been demonstrated by AHR knockout studies in which AHR knockout mice do not show typical dioxin-induced toxicity compared with their AHR-expressing littermates (Fernandez-Salguero et al. 1996). There is evidence that some of the toxicity of these pHAHs may be directly due to CYP1A activity; for example, CYP1A2 knockout mice are resistant to liver damage and uroporphyria when exposed to TCDD (Smith et al. 2001). And male CYP1A1 knockout mice are protected against TCDD-mediated lethality and wasting syndrome (Uno et al. 2004b). Furthermore, Cantrell et al. (1996) were able to reduce TCDD-induced DNA degradation and damage to the medial yolk vein in medaka by cotreating the embryos with the P450 inhibitor piperonyl butoxide (PBO). Dong et al. (2002) found that cotreatment of zebrafish embryos with the partial AHR antagonist and CYP1A inhibitor α-naphthoflavone (ANF) or the P450 inhibitors SKF525A or miconazole reversed the reduction of blood flow in the mesencephalic vein and midbrain apoptosis caused by TCDD. Another study by Teraoka et al. (2003) showed that a morpholino knockdown of CYP1A and AHR2 in zebrafish prevented the pericardial edema and trunk circulation failure caused by TCDD.

Although there is a strong, positive relationship between the ability of PAHs to bind the AHR and their induction of CYP1A (Billiard et al. 2002), conclusions regarding the role of the AHR pathway and CYP1A activity in the toxicity of PAHs have been less clear. In a mammalian study, homozygous CYP1A1 knockout mice showed less liver damage and survived the acute effects of injection of the PAH benzo(*a*)pyrene (BaP) for 3 days longer than did those that were heterozygous for CYP1A1 (Uno et al. 2001). However, these CYP1A1 knockout mice also showed 4-fold higher levels of BaP–DNA adducts than did those heterozygous for CYP1A1. This study suggests that acute lethality of BaP was reduced by lack of CYP1A1 but that genotoxicity was actually increased by the lack of CYP1A1 (Uno et al. 2001). In a recent study by this group, BaP administered in the diet caused lethality in CYP1A1 knockout mice at a dose that was not lethal to CYP1A1-expressing mice (Uno et al. 2004a). These authors suggested that rather than CYP1A1 activity enhancing the toxicity of BaP, as has been previously suggested, CYP1A1 is critical for the detoxication of orally administered BaP in mice.

Billiard (2002) compared a variety of PAHs with various affinities for the AHR and potencies for CYP1A induction in juvenile rainbow trout; chemicals ranged from the strong CYP1A inducer benzo(*k*)fluoranthene, to the relatively weak, alkylated inducer retene and the noninducer phenanthrene. Billiard (2002) found that the rank order for CYP1A induction in these fish did not predict the rank order for the induction of blue-sac-like symptoms; in fact, the only PAHs that caused blue-sac-like symptoms were retene and phenanthrene, the low- and non-inducing PAHs used in that study. Hawkins et al. (2002) observed apparent additive toxicity in juvenile and larval rainbow trout coexposed to one of two PAHs, the alkylated AHR agonist retene or the non-AHR-agonist phenanthrene, with the P450 inhibitor PBO. In contrast, another study found that cotreatment with the partial AHR antagonist and CYP1A inhibitor ANF prevented the reduction of circulation in the dorsal midbrain of zebrafish caused by the PAH-type AHR agonist β-naphthoflavone (BNF; Dong et al. 2002). From these studies, it is clear that the relationship between CYP1A activity and PAH toxicity is complex and that reduced CYP1A activity is sometimes, but not always, protective of PAH toxicity.

In an attempt to *a*) clarify the role of CYP1A activity in the toxicity of PAHs and *b*) explore the possible effects of co-occurring PAH-type CYP1A inducers and inhibitors, we cotreated *Fundulus heteroclitus* (killifish) embryos with three different AHR agonists [the pHAH 3,3′,4,4′,5-pentachlorobiphenyl (PCB-126) and the PAHs BNF and BaP] and four CYP1A inhibitors that work by various mechanisms (Table 1). The compounds here collectively referred to as CYP1A inhibitors have all been shown to inhibit CYP1A activity (see references in Table 1); however, the specificities of these CYP1A inhibitors for CYP1A over other P450s in our system are not known. These inhibitors included the aforementioned model compounds ANF and PBO and the environmentally relevant hydrocarbons fluoranthene (FL) and 2-aminoanthracene (AA) (Watson et al. 1995; Willett et al. 1998, 2001). We then observed embryos for *in ovo* CYP1A activity, as measured by ethoxyresorufin-*O*-deethylase (EROD) activity, and for deformities, including pericardial edema, heart elongation, cranial–facial malformations, and tail abnormalities. In these experiments, we used a wide range of concentrations of AHR agonists to elicit a range of EROD inductions with and without inducing deformities; concentrations of inhibitors were selected with the goal of eliciting the maximal inhibition of EROD without inducing deformities. Our results indicate that coexposure to PAH-type AHR agonists and CYP1A inhibitors consistently enhanced embryotoxicity beyond levels predicted by an additive toxicity model.

## Materials and Methods

### Reagents.

BaP, BNF, ANF, FL, AA, PBO, and ethoxyresorufin were purchased from Sigma Aldrich (Saint Louis, MO). PCB-126 was purchased from Chem Service (West Chester, PA). Dimethyl sulfoxide (DMSO) and acetone were purchased from Mallinckrodt Baker (Phillipsburg, NJ).

### Fish care.

Adult killifish were captured with minnow traps from King’s Creek, Virginia (a well-characterized reference site with low sediment PAH levels; Mulvey et al. 2002) and transported to the Ecotoxicology Laboratory of Duke University. Fish were maintained in 70-L or 100-L aquaria at 24°C with a 16-hr light/8-hr dark cycle and were fed TetraMin flakes (Tetra Sales, Blacksburg, VA) *ad libitum*. Fish were held in laboratory conditions for at least 3 weeks before embryo acquisition. Embryos were obtained from *in vitro* fertilization of pooled oocytes stripped from 9–12 females with pooled milt from 4–5 males.

### In ovo *EROD.*

**Figure 1:**
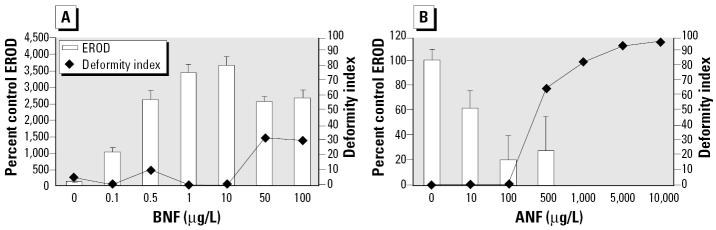
Dose–response curves showing percent control *in ovo* EROD induction and deformity index in embryos exposed to (*A*) BNF or (*B*) ANF. EROD values are missing for the 1,000, 5,000, and 10,000 μg/L concentrations because embryos from these treatment groups were too deformed to score for *in ovo* EROD. For the BNF control group, *n* = 20; for all other BNF treatments, *n* = 9 or 10. For each ANF treatment group, *n* = 8–10. EROD values are mean ± SEM. See “Results” for explanation of statistical differences.

We used an *in ovo* EROD method, modified slightly from the method described by Nacci et al. (1998, in press), to measure the CYP1A activity of embryos. Several hours after fertilization, embryos with dividing cells were selected and placed individually in 20-mL scintillation vials with 10 mL artificial seawater (20 parts per thousand; Instant Ocean, Mentor, OH) containing 21 μg/L ethoxyresorufin with or without an EROD inducer (BNF, BaP, or PCB-126) and/or an EROD inhibitor (ANF, AA, FL, or PBO). We used either acetone or DMSO as the solvent, and solvent concentrations were < 0.015% for all treatments except the high doses in the ANF-alone dose group (Figure 1), in which solvent concentrations were ≤0.1%. Embryos were in dosing solution for 7 days, during which resorufin, the fluorescent product of CYP1A metabolism of ethoxyresorufin, accumulated in the embryos’ bi-lobed urinary bladders. On day 7 of development, embryos were placed in clean artificial seawater, and embryo bladders were visualized by fluorescent microscopy (50× magnification using rhodamine red filter set; Axioskop; Zeiss, Thornwood, NY). EROD activity was measured as intensity of the bladder fluorescence and was quantified digitally by IPLab software (Scanalytics Inc., Fairfax, VA). *In ovo* EROD values were expressed as a percentage of control intensity. Individuals with deformed bladders or with fluorescence in areas other than the bladder (e.g., the pericardial sac in some embryos with severe pericardial edema) were excluded from *in ovo* EROD measurement. Although ethoxyresorufin has been shown to be nondetrimental to embryos (Nacci et al. 1998), coexposures of ANF and BNF were done with and without ethoxyresorufin to rule out a possible interactive effect of the ethoxyresorufin. No differences were observed between the deformities of embryos with or without ethoxyresorufin (data not shown).

### Deformity assessment.

Embryos were scored blind for heart elongation (tube heart), pericardial edema, tail shortening, and hemorrhaging on day 10 of development. Heart deformities were found to be the most sensitive end point scored, so this end point was used for further analysis. Heart elongation severity was ranked between 0 and 5, and a deformity index for each treatment was calculated as sum of scores for individuals in that treatment group divided by the maximum score possible (the number of individuals multiplied by 5). This quotient was then multiplied by 100.

### Experimental approach.

Embryos were exposed to nominal concentrations of one of three AHR agonists alone and in combination with nominal concentrations of one of four CYP1A inhibitors. We used the AHR agonists PCB-126, BNF, and BaP (Table 1). BNF and BaP were chosen as model PAH-type AHR agonists. BNF is a synthetic compound, commonly used as a model AHR agonist in studies, whereas BaP is a naturally occurring PAH, commonly found in environmental mixtures. We chose PCB-126 as a model pHAH-type AHR agonist.

**Figure 2:**
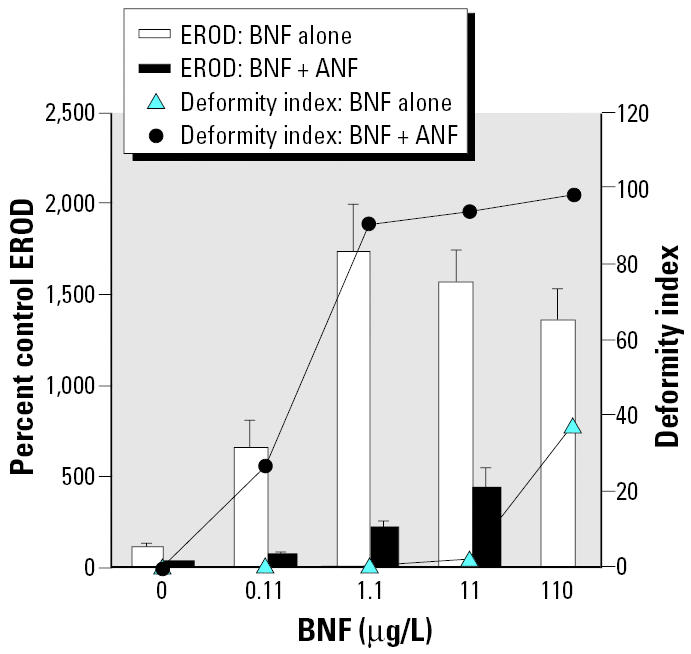
Effects of BNF with and without 100 μg/L ANF cotreatment on *in ovo* EROD and deformity index. The EROD value is missing for the 110 μg/L BNF + ANF treatment group because embryos in this treatment group were too deformed to score for *in ovo* EROD; *n* = 8 or 9 for each treatment group, except for EROD measurement in the 1.1 μg/L BNF + ANF (*n* = 6) and 11 μg/L BNF + ANF (*n* = 2) treatment groups, because the remainder of embryos were too deformed to score for *in ovo* EROD. EROD values are mean ± SEM. See “Results” for explanation of statistical differences.

We used the inhibitors ANF, PBO, FL, and AA in this study; their mechanisms of actions are listed in Table 1. We chose ANF because it is well characterized for its activities as both a partial AHR antagonist (Merchant et al. 1990, 1992) and a competitive CYP1A inhibitor (Goujon et al. 1972; Testa and Jenner 1981). BNF and ANF dose–response curves were first established using a range of concentrations and scoring for deformities and *in ovo* EROD (Figure 1). Subsequently, coexposures were performed using a range of BNF concentrations that spanned concentrations found to induce EROD, but not deformities, to concentrations that caused both EROD induction and deformities, with a concentration of ANF (100 μg/L) that dramatically lowered *in ovo* EROD measurements but did not by itself cause deformities (Figure 2).

**Figure 3:**
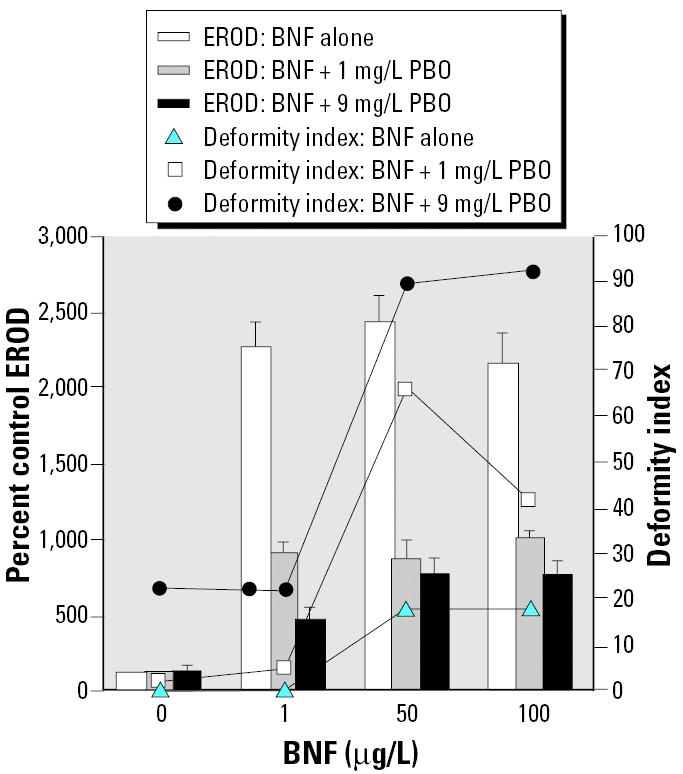
Effects of BNF with and without 1 or 9 mg/L PBO cotreatment on *in ovo* EROD and deformity index; *n* = 7–10 for each treatment group, except for EROD measurements in the 50 μg/L BNF + 9 mg/L PBO (*n* = 5) and 100 μg/L BNF + PBO (*n* = 6) treatment groups, because the remainder of embryos were too deformed to score for *in ovo* EROD. EROD values are mean ± SEM. See “Results” for explanation of statistical differences.

In order to distinguish between the effects of AHR antagonism and CYP1A inhibitory effects, both of which occur with ANF exposure, we also used the P450 inhibitor PBO. PBO is a quasi-irreversible P450 inhibitor that acts by forming a metabolic intermediate complex with the heme group of P450 enzymes, thereby preventing the redox cycling of the enzyme (Hodgson and Philpot 1974; Testa and Jenner 1981). We cotreated embryos with a range of BNF concentrations (1–100 μg/L) and either 1 or 9 mg/L PBO (Figure 3).

**Figure 4:**
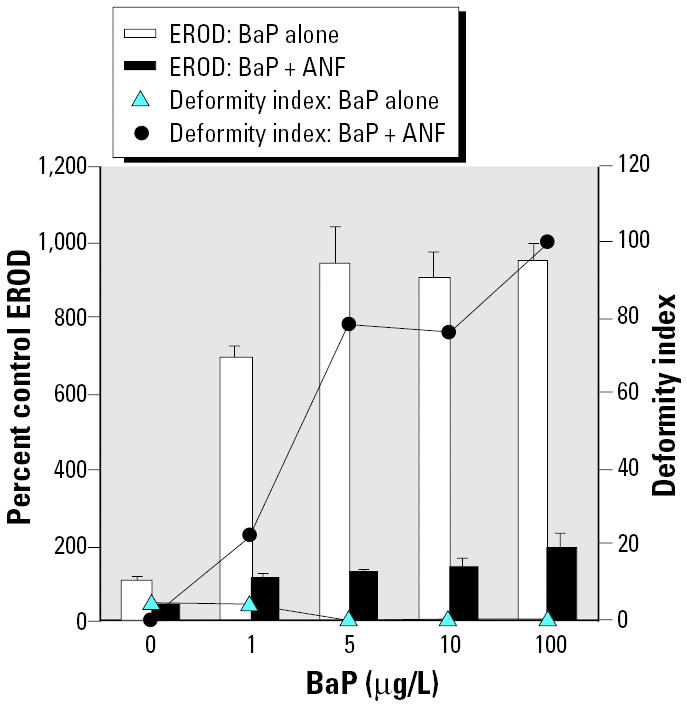
Effects of BaP with and without 100 μg/L ANF cotreatment on *in ovo* EROD and deformity index; *n* = 9 or 10 for each treatment group, except for EROD measurement in the 5 μg/L BaP + ANF (*n* = 7), 10 μg/L BaP + ANF (*n* = 7), and 100 μg/L BaP + ANF (*n* = 3) treatment groups, because the remainder of embryos were too deformed to score for *in ovo* EROD. EROD values are mean ± SEM. See “Results” for explanation of statistical differences.

To test the effects of EROD inhibition on embryos coexposed to an environmentally relevant AHR agonist, BaP and ANF coexposures were conducted. In this experiment the ANF concentration was 100 μg/L, a concentration previously established as effective at lowering *in ovo* EROD without inducing deformities. BaP concentrations ranged from 1 to 100 μg/L (Figure 4).

**Figure 5:**
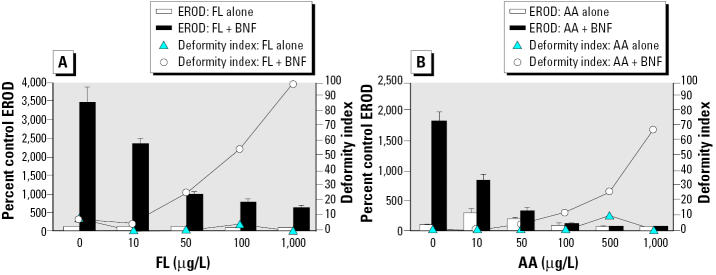
Effects of FL (*A*) and AA (*B*) with and without 1 μg/L BNF cotreatment on *in ovo* EROD and deformity index. For (*A*), *n* = 9 or 10 for each treatment group, except for EROD measurement in the 1 μg/L BNF + 1,000 μg/L FL treatment group (*n* = 4), because the remainder of embryos were too deformed to score for *in ovo* EROD. For (*B*), *n* = 16 and 19 for control and BNF-alone treatment groups, respectively; for other treatment groups, *n* = 6–10. EROD values are mean ± SEM. See “Results” for explanation of statistical differences.

To test the effectiveness of environmentally relevant PAHs at inhibition of *in ovo* EROD and to determine how inhibition by these compounds affected deformities, embryos were exposed to a range of FL and AA concentrations alone and with 1 μg/L BNF (Figure 5).

**Figure 6:**
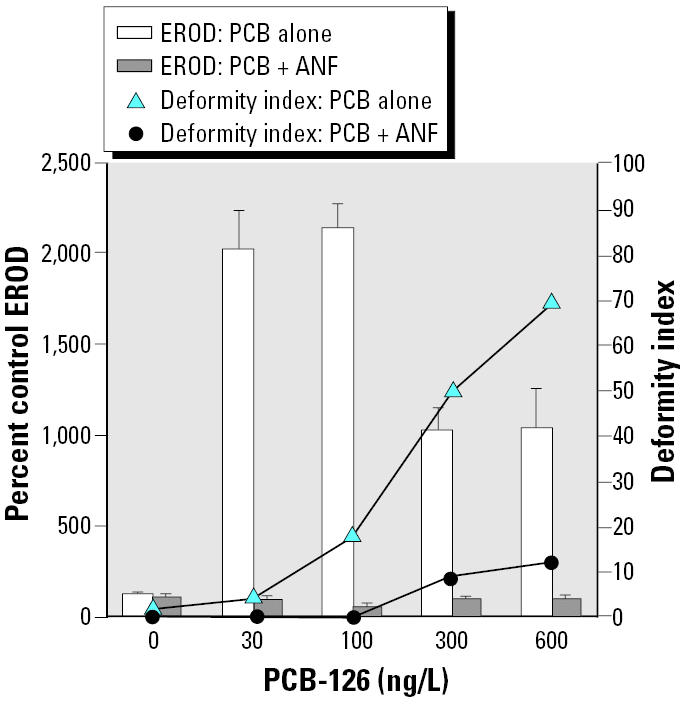
Effects of PCB-126 with and without 100 μg/L ANF cotreatment on *in ovo* EROD and deformity index; *n* = 9 or 10 for all treatment groups. EROD values are mean ± SEM. See “Results” for explanation of statistical differences.

In order to assess interactions between a representative pHAH and a CYP1A inhibitor in killifish, embryos were exposed to concentrations of PCB-126 that spanned from concentrations known to induce EROD that cause low-deformity indices, to concentrations that induce severe deformities, with and without 100 μg/L ANF (Figure 6).

### Data analysis and representation.

Data were analyzed using Statview for Windows (Version 5.0.1; SAS Institute Inc., Cary, NC). EROD values were analyzed by one- and two-way analysis of variance (ANOVA). When ANOVA yielded significance (*p* < 0.05), Fisher’s protected least-significant differences was used as a post hoc test. Deformity data were ordinal in nature and were therefore assessed using rank order tests—the Mann-Whitney *U*-test for analyses with two variables and the Kruskal-Wallis test for analyses with three or more variables. *p*-Values corrected for ties in rank are reported for these analyses as “tied *p*-values.” Each graph represents a separate experiment. Although deformities were analyzed statistically using individual severity rankings, deformity data are shown as a deformity index for clarity. Interactions were characterized as synergistic based on significance of a one-group chi square analysis comparing the observed frequencies of deformities with frequencies predicted by an additive interaction (calculated as a sum of the deformity frequency for each treatment; predicted frequency had minimum value of 1 for this analysis because chi square calculation requires predicted frequency in the denominator of an equation).

## Results

Embryos dosed with BNF alone showed *in ovo* EROD induction at all concentrations tested (*p* ≤0.0002) that was maximal at the 10 μg/L concentration (Figure 1A). At 50 and 100 μg/L, EROD activities declined to below the maximal level (*p* = 0.0001 and 0.0003, respectively). Coincident with this decline, embryos exposed to 50 and 100 μg/L BNF exhibited elevated deformity indices (effect of BNF on deformities, tied *p* < 0.0001). Embryos exposed to 10, 100, and 500 μg/L ANF alone displayed lower EROD activities than controls (Figure 1B; *p* < 0.0001). Embryos exposed to ANF levels > 500 μg/L were too deformed to allow for measurement of *in ovo* EROD. Embryos exposed to 10 μg/L ANF and 100 μg/L ANF exhibited no deformities, whereas those exposed to ≥500 μg/L ANF exhibited high deformity indices (effect of ANF on deformities, tied *p* < 0.0001).

In a separate experiment designed to explore the interaction between ANF and BNF coexposures, embryos were dosed with a range of BNF concentrations with or without 100 μg/L ANF (Figure 2), the dose of ANF shown to be most effective in inhibiting EROD without causing deformities by itself (Figure 1). Embryos exposed to BNF alone exhibited significant EROD induction at all concentrations (*p* < 0.0001). Cotreatment with ANF significantly inhibited *in ovo* EROD activities (*p* < 0.0001). Embryos cotreated with ANF and 110 μg/L BNF were too deformed for *in ovo* EROD measurements. In embryos treated with BNF alone, deformities were noted only at the 110 μg/L concentration (effect of BNF alone on deformities, tied *p* = 0.0011). However, ANF-cotreated embryos were deformed at all BNF concentrations. That is, embryos were deformed at BNF concentrations three orders of magnitude lower when BNF treatment was combined with 100 μg/L ANF than when treated with BNF alone (overall effect of BNF and ANF on deformities, tied *p* < 0.0001 for each).

In an experiment exploring the effect of cotreatment of embryos with BNF and PBO (Figure 3), all BNF concentrations significantly induced *in ovo* EROD activities (*p* < 0.0001). Cotreatment with both concentrations of PBO (1 and 9 mg/L) lowered *in ovo* EROD across all BNF concentrations (*p* < 0.0001). Embryos exposed to PBO at the low concentration had very low deformities that were not statistically different from controls (tied *p* = 0.3173). Embryos exposed to the high concentration of PBO had an elevated deformity index (effect of PBO alone on deformities, tied *p* = 0.0448). Coexposures to BNF and PBO caused increased deformity indices over those seen in embryos dosed with BNF alone or PBO alone at all BNF concentrations (overall effect of BNF and PBO on deformities, tied *p* < 0.0001 and = 0.0021 respectively).

We also examined a range of concentrations (1–100 μg/L) of BaP, an environmentally relevant PAH, with and without coexposure to 100 μg/L ANF (Figure 4). BaP alone significantly induced EROD at all doses tested (*p* < 0.0001), and ANF cotreatment lowered the *in ovo* EROD activity (*p* < 0.0001). Embryos dosed with BaP alone exhibited low deformity indices that were not statistically different from controls (effect of BaP alone on deformities, *p* = 0.1856), whereas those dosed with BaP in combination with 100 μg/L ANF had elevated deformity indices at all BaP concentrations tested (overall effect of ANF on deformities, tied *p* < 0.0001).

Exposure to environmentally relevant CYP1A inhibitor FL by itself caused *in ovo* EROD activities below control levels (*p* < 0.0001; Figure 5); however, when embryos were coexposed to FL with 1 μg/L of the inducer BNF, EROD activities were induced (*p* < 0.0001) and there was an FL-dose–dependent decrease in *in ovo* EROD activities (*p* < 0.0001). Embryos exposed to FL alone did not exhibit elevated deformity indices (tied *p* = 0.3764); BNF at this concentration also did not cause an elevated deformity index (effect of BNF alone on deformities, tied *p* = 0.1681). However, when FL exposure was combined with 1 μg/L BNF, high deformity indices were observed at FL levels of ≥50 μg/L (overall effect of FL on deformities, tied *p* = 0.0002; overall effect of BNF on deformities, tied *p* < 0.0001).

Exposure to AA alone elicited slight EROD induction at the 10 and 50 μg/L concentrations (*p* < 0.0001 and *p* = 0.0163, respectively; Figure 5); however, when embryos were coexposed to 1 μg/L BNF, the BNF-mediated EROD induction was inhibited in a dose-dependent fashion by increasing AA concentrations (*p* < 0.0001). Embryos dosed with AA alone exhibited low deformity indices (not significant, tied *p* = 0.6609), but when embryos were coexposed to AA with 1 μg/L BNF, deformity indices were elevated in cotreatments of BNF with AA concentrations of ≥50 μg/L (overall effect of AA and BNF on deformities, tied *p* < 0.0001 for each).

The pHAH PCB-126 significantly induced *in ovo* EROD over controls at all doses tested (*p* < 0.0001; Figure 6). Concentrations of 300 and 600 ng/L induced EROD levels less than the maximal levels achieved by 30 and 100 ng/L (*p* < 0.0001 for each). Deformity indices were elevated in embryos exposed to PCB-126 concentrations of ≥100 ng/L (effect of PCB-126 alone on deformities, tied *p* > 0.0001). In the case of PCB-126, however, coexposure with 100 μg/L ANF dramatically decreased the deformity indices of PCB-126 treatment groups (overall effect of PCB-126 and ANF on deformities, tied *p* < 0.0001 and = 0.0003, respectively).

Synergistic interactions, determined by one-group chi square analyses, yielded deformity frequencies greater than predicted additive values for cotreatments with BNF + ANF, BaP + ANF, BNF + FL, and BNF + AA (*p* < 0.001 for each; Figures 2, 4, and 5, respectively). The interaction for BNF + 1 mg/L PBO cotreatment approached significance (*p* = 0.051); however, BNF + 9 mg/L PBO cotreatment was not synergistic (Figure 3).

## Discussion

The results of this study demonstrate that the embryotoxicity of the pHAH PCB-126 was decreased with coexposure to the CYP1A inhibitor and AHR antagonist ANF. This result is in general agreement with other studies showing the reduction of early-life-stage toxicity of pHAHs when CYP1A activity or AHR-mediated signaling was decreased (Cantrell et al. 1996; Dong et al. 2002; Teraoka et al. 2003). In contrast, in the present study the embryotoxicities of two PAH-type AHR agonists were increased when CYP1A was inhibited by chemicals that act by various modes of action. The data for the interactions between the PAH-type inducers and inhibitors clearly indicate a synergistic effect on embryotoxicity for coexposures to BNF + ANF, BaP + ANF, BNF + FL, and BNF + AA. The BNF + 1 mg/L PBO dose was nearly significant for synergism (*p* = 0.051).

The various inhibitors used in this study caused similar increases in PAH toxicity, although these inhibitors varied in structure and mechanism of inhibition. This suggests that the increased toxicity of PAHs by CYP1A inhibitors is due to the shared characteristic of CYP1A inhibition and is not specific for a particular structure or mechanism of inhibition. The PAH interactions with CYP1A inhibitors observed in this study are in general agreement with those found in a previous study in which we showed that an extract from a site highly contaminated with PAHs was more toxic when coexposed with several CYP1A inhibitors (Wassenberg and Di Giulio 2004).

Although the pHAH PCB-126 and the PAHs BNF and BaP share the characteristic of being AHR agonists, the difference between the effect of CYP1A inhibition in the pHAH-versus the PAH-dosed embryos is striking. This difference may be due to the fundamentally different chemistries and somewhat different toxicities of these two classes of compounds. PCBs and other halogenated compounds are relatively stable, long-lived compounds. Although pHAHs induce mono-oxygenases such as CYP1A, metabolism of these compounds is relatively slow (White et al. 1997). The half-life of PCB-126 administered to juvenile rainbow trout in their diet was found to be between 82 and 180 days (Brown et al. 2002). In contrast, PAHs are rapidly metabolized. Half-lives of nine PAHs orally administered to adult rainbow trout were estimated to be ≤9 days (Niimi and Palazzo 1986). *In vitro* metabolism of BaP was found to be 2,000–4,000 times faster than metabolism of the coplanar pHAH PCB-77 in induced scup (*Stenotomus chrysops*) microsomes (Stegeman et al. 1981; White et al. 1997). This rapid metabolism of PAHs allows for more rapid excretion of the compound but can also activate PAHs into more reactive intermediates that can bind to and damage cellular constituents. Studies of PAH metabolism by fish embryos are very limited. However, Fong et al. (1993) demonstrated extensive phase 1 and phase 2 metabolism of 7,12-dimethylbenz(*a*)anthracene by rainbow trout embryos. Additionally, the presence and inducibility of CYP1A in killifish embryos observed in this and previous studies (Meyer et al. 2002; Nacci et al. 1998; Toomey et al. 2001) support the hypothesis that PAH metabolism is occurring in embryos in the present study. Therefore, it is possible that inhibition of CYP1A in the PAH-treated embryos extended the half-life of the PAH, causing prolonged AHR agonism, similar to AHR agonism in pHAH-treated animals.

Some PAHs act through a narcotic mechanism in which the compounds accumulate in tissues to a level at which they physically interfere with membranes (McCarty and Mackay 1993). The inclusion of a CYP1A inhibitor with PAHs would be likely to slow metabolism of the PAHs. However, it is not likely that narcosis is responsible for the synergy observed in these experiments. First, even if the total amount of compound to which the embryos were exposed in deformed treatment groups accumulated within the embryo, the concentration of PAH would not reach the 2–8 mmol/kg threshold for acute narcosis (McCarty and Mackay 1993). Second, narcotic modes of action are, by definition, additive, and an additive model of toxicity does not fit our data.

It has been suggested that the toxicity of pHAHs is at least in part tied to an oxidative stress mode of damage (Nebert et al. 2000; Stohs 1990). The pHAHs fit into the active site for CYP1A but are poor substrates for CYP1A metabolism, causing an uncoupling of electron flow between the enzyme and the substrate. This uncoupling, together with increased expression of CYP1A via the AHR, is believed to lead to the production of reactive oxygen and oxidative damage (Schlezinger and Stegeman 2001; Schlezinger et al. 1999; Shertzer et al. 2004). We included PBO as an inhibitor in our studies because it binds to the heme group of P450s, thereby inhibiting electron flow from the enzyme and preventing this uncoupling. Because PBO enhanced toxicity in PAH-cotreated embryos, P450 uncoupling is not supported as the mechanism underlying the interactive toxicity of PAHs and CYP1A inhibitors observed in this study.

However, other mechanisms of oxidative stress may play a role in PAH-driven toxicity. An oxidative stress mechanism for the toxicity of the alkylated PAH retene has been proposed based on reduced ratios of glutathione to glutathione disulfide (GSH:GSSG) in rainbow trout larvae at retene exposures that exhibited blue-sac-like symptoms (Billiard 2002). Many PAHs (including BaP) can be metabolized to quinones (Bolton et al. 2000). These reactive metabolic intermediates are capable of further AHR agonism, redox cycling, and generation of reactive oxygen species, which can then perturb cellular redox status and damage macromolecules and are cytotoxic and mutagenic (Bolton et al. 2000; Burezynski and Penning 2000). The metabolism of PAHs to reactive compounds is clearly associated with their genotoxicity and carcinogenicity (Levin et al. 1982; Sjögren et al. 1996). Inhibition of CYP1A would likely alter the metabolism of PAHs, possibly generating more embryotoxic intermediates. However, the extent to which altered metabolism affected the PAH toxicity observed in this study is not known. Current studies are addressing mechanisms underlying the interactive toxicities reported herein.

### Importance of findings.

PAH contamination levels are increasing in aquatic systems across the United States (Van Metre et al. 2000). Sites with PAH mixtures generally contain agonists for the AHR that can induce CYP1A activity, such as BaP, chrysene, and benzo(*k*)fluoranthene. These mixtures may also contain compounds that can act as CYP1A inhibitors. The noncompetitive CYP1A inhibitor FL, for example, is one of the more prevalent PAHs found in marine sediments, lakes, and rainwater (Latimer and Zheng 2003; Van Metre et al. 2000). Aminoanthracenes are components in coal liquefaction products (Pelroy and Wilson 1981; Wilson 1980) and may also be found in environmental mixtures. It is possible that other compounds found in environmental mixtures may also be as yet uncharacterized CYP1A inhibitors. The synergisms found in this study indicate that compounds such as BaP, FL, and AA, which can be commonly found in environmental mixtures, may be substantially more toxic in their mixtures than an additive approach to PAH toxicity would predict, and that additive models currently used to estimate PAH toxicity (e.g., Barron et al. 2004; Di Toro et al. 2000) may underestimate the toxicity of PAH mixtures. Additionally, the observed end point for this synergy was cardiovascular development during early development, a sensitive life stage for vertebrates in general.

## Figures and Tables

**Table 1:** AHR agonists and CYP1A inhibitors used in this study.

Compound	Type	Structure	Mechanism of action	Sample references
AHR agonists				
BNF	Synthetically derived model PAH	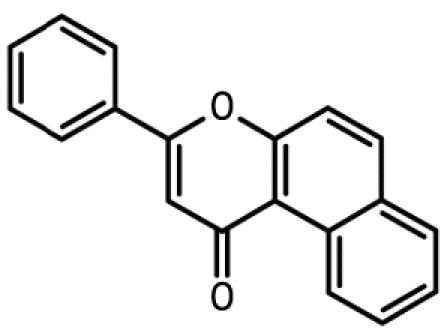		Matsuda et al. 1995; Ronisz and Förlin 1998
BaP	Environmentally relevant PAH	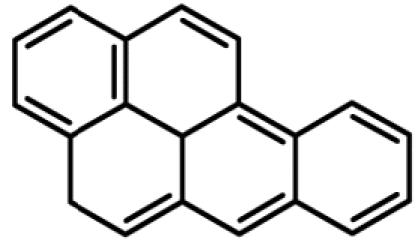		Chaloupka et al. 1993; Fent and Bätscher 2000; Van Veld et al. 1997
PCB-126	Environmentally relevant pHAH	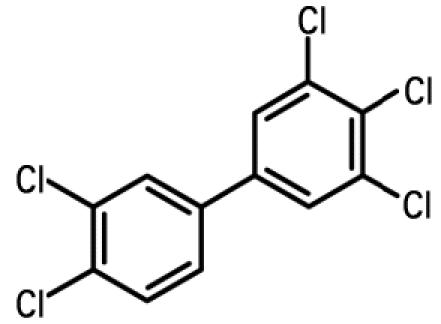		Abnet et al. 1999; Dabrowska et al. 2000
CYP1A inhibitors				
ANF	Synthetically derived model PAH	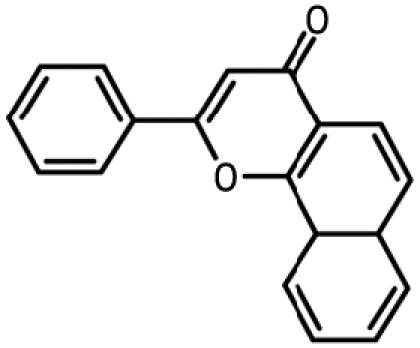	Partial AHR antagonist and competitive inhibitor of CYP1A	Goujon et al. 1972; Lu et al. 1996; Merchant et al. 1990, 1992, 1993; Merchant and Safe 1995; Miranda et al.1998; Testa and Jenner 1981
PBO	Methlenedioxybenzene derivative	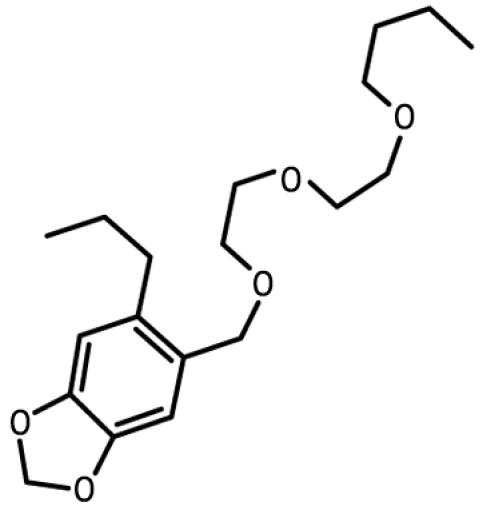	P450 inhibitor; forms a metabolic intermediate with heme group of P450	Hodgson and Philpot 1974; Miranda et al. 1998; Murray and Reidy 1990; Testa and Jenner 1981
FL	Environmentally relevant PAH	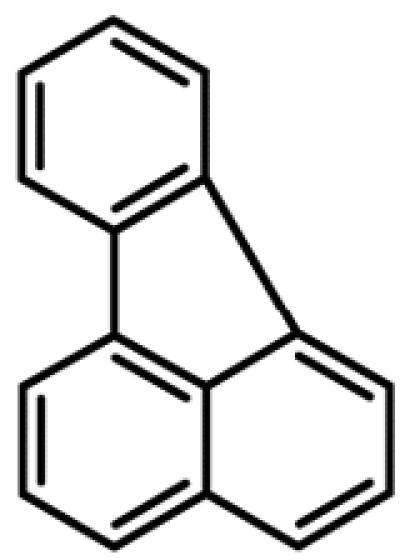	Competitive inhibitor of CYP1A *in vitro;* modestly lowers CYP1A protein expression *in vivo*	Willett et al. 1998, 2001
AA	Environmentally relevant aromatic amine	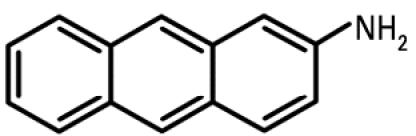	Mechanism-based CYP1A inhibitor; binds to CYP1A and causes its degradation	Watson et al. 1995
